# Measurement and Analysis of Near-Ground Propagation Models under Different Terrains for Wireless Sensor Networks

**DOI:** 10.3390/s19081901

**Published:** 2019-04-22

**Authors:** Weisheng Tang, Xiaoyuan Ma, Jianming Wei, Zhi Wang

**Affiliations:** 1 Shanghai Advanced Research Institute, Chinese Academy of Sciences, Shanghai 201210, China; tangws@sari.ac.cn (W.T.); maxy@sari.ac.cn (X.M.); wjm@sari.ac.cn (J.W.); 2 University of Chinese Academy of Sciences, Beijing 100049, China; 3 State Key Laboratory of Industrial Control Technology, Zhejiang University, Hangzhou 310027, China

**Keywords:** Wireless sensor networks (WSNs), near-ground propagation model, path loss, terrain factor

## Abstract

The propagation model is an essential component in the design and deployment of a wireless sensor network (WSN). Although much attention has been given to near-ground propagation models, few studies place the transceiver directly on the ground with the height of antennas at the level of a few centimeters, which is a more realistic deployment scenario for WSNs. We measured the Received Signal Strength Indication (RSSI) of these truly near-ground WSNs at 470 MHz under four different terrains, namely flat concrete road, flat grass and two derived scenarios, and obtained the corresponding path loss models. By comprehensive analysis of the influence of different antenna heights and terrain factors, we showed the limit of existing theoretical models and proposed a propagation model selection strategy to more accurately reflect the true characteristics of the near-ground wireless channels for WSNs. In addition, we implemented these models on Cooja simulator and showed that simplistic theoretical models would induce great inaccuracy of network connectivity estimation.

## 1. Introduction

A wireless sensor network (WSN) is composed of sensor nodes that are able to communicate with each other through wireless channels [1,2]. With the growth of research on WSNs in both academia and industry, WSNs have been used in a wide variety of applications, such as infrastructure monitoring, industrial control, environmental sensing, and surveillance. An increasing number of practical applications prompts WSN research to be more realistic in every aspect. As with any other wireless communication system, the propagation model is an essential and critical tool for WSN planning and deployment [3]. The propagation model describes the radio channel characteristics, which are closely linked with the environment and physical parameters. By predicting the attenuation and distortion of the radio-frequency signal, the expected received signal strength level can be calculated. Many studies reveal that there is a complex relationship among the propagation characteristic, the sensor placement, and the network connectivity [4]. Most existing research on WSNs implies the basic assumption of accurate propagation models. However, wireless channels can be easily affected by interference, noise and other factors, and the fading characteristics are closely related to the communication environment. The WSN would not perform as expected under realistic scenarios if simplistic or idealized propagation models are adopted. Since WSNs are often deployed in specific areas to monitor various environmental indicators in real-time [5], especially in harsh areas for emergency applications, an appropriate propagation model based on specific environment and terrain is of the utmost importance to adequately predict the link quality and coverage of WSNs.

Most previous studies of radio propagation models assume that antennas are placed at least 0.5 m above the ground [6]. However, in most WSN applications, sensors are thrown directly on the ground. Hence, existing propagation models cannot correctly reflect the real characteristics of these truly near-ground wireless channels.

In one of the early studies of near-ground propagation characteristics [7], the authors investigated short-range near-ground scenarios using a two-ray model, and noticed a significant decrease in signal strength as transceivers get closer to the ground. Joshi et al. [8] showed results from narrowband and wideband measurements conducted at 300 MHz and 1900 MHz investigating effects of transmitting and receiving antenna heights, antenna radiation patterns, and effects of foliage in a forest environment, but the antenna height was set to 0.75–1.55 m, which is not close enough to ground. In [9], the near-ground propagation channel measurements at 868 MHz in three different scenarios are implemented. In [10], outdoor measurement and modeling of three near-ground sites are conducted at 2.4 GHz frequency, and the prediction accuracy of one- and two-slope models are compared. In [11], the authors obtained the path loss models of outdoor and indoor scenarios at 3.5 GHz, using simple one-slope model, with antenna heights of 0.3 m and 2.1 m, respectively. The effect of vegetation barriers is also taken into consideration. Kurt and Tavli [12] listed some constraints when exploring WSN specific propagation models, such as low antenna heights, directivity of antennas, low transmission power and network topology, where the authors briefly introduced five optional propagation models for near-ground WSNs. Sangodoyin et al. [13] measured characteristics of the outdoor near-ground propagation, and found that the path loss shows crucial dependence on antenna heights. The authors indicated that the path loss has slight dependence on frequency. Other studies (e.g., [14,15,16]) propose empirical path loss models for different outdoor environments, and confirm that the type of terrain can have a significant impact on radio propagation near ground. Although much attention has been given to near-ground propagation models, few studies place the transceiver directly on the ground, with the antenna height at the level of a few centimeters, which is a more realistic deployment scenarios for WSNs.

Studying the actual performance of protocols through field test-beds requires a tremendous amount of work [17]. Therefore, various simulation tools are used to expediently test and evaluate new protocols. However, these simulation results are strongly influenced by the selected propagation models. Most existing simulators adopt simplistic theoretical models such as Free Space Path Loss (FSPL) model and two-ray model [18]. Although simple models perform well in some cases, unrealistic models with unreasonable physical layer assumptions may lead to unreliable simulation results, which are far from practical applications.

We measured the received signal strength indication (RSSI) of several near-ground wireless channels at 470 MHz, with four terrains and three antenna heights. Through comprehensive studies, we derived several statistic propagation models to more accurately reflect the characteristics of near-ground path loss. We also analyzed the impact of antenna height and terrain, and further investigated the propagation model selection strategy in different scenarios. Furthermore, we explored the impact of near-ground propagation models on network connectivity by simulations.

The contributions of this paper can be summarized as follows.

1. We derived several statistic propagation models to describe near-ground path loss in four terrains, including two derived scenarios with different terrain factors.
2. We analyzed the impact of antenna height and terrain factors on the radio path loss at ground level, and further investigated the propagation model selection strategy in near-ground scenarios.
3. We implemented the measured propagation models on Cooja simulator [19] and showed that simplistic theoretical models induce great deviation of network connectivity.

The rest of this paper is organized as follows. Section 2 describes some popularly used WSN propagation models. Section 3 describes the measurement setup. Section 4 shows the measurement results and analyzes the influence factors of propagation models. Section 5 discusses the impact of path loss models on network connectivity in the simulations. Finally, Section 6 concludes the paper.

## 2. WSN Propagation Models

The wireless channel refers to the transmitter antenna, the receiver antenna, and the propagation path between them. Radio channels use electromagnetic waves to transmit signals, and the main forms of radio wave propagation are space waves, including direct wave, refracted wave, scattered wave, and their synthesis wave [20]. A propagation model is a set of mathematical expressions or diagrams which can describe the radio characteristics and estimate the signal strength drop. It can be estimated either by theoretical modeling or based on direct experimental measurements. The free-space model [21] is the most widely used model. The model is based on the Friis transmission formula, which indicates that, in an ideal environment, the power is spread uniformly over the surface of a sphere surrounding the antenna. The free space path loss (FSPL) can be defined as

(1) $${P\;L}_{F}\;=\;\frac{P_{t}}{P_{r}}G_{t}G_{r}\;=\;{\left(\frac{4\pi d}{\lambda }\right)}^{2}$$

where $P_{t}$ and $P_{r}$ are the transmitted and received power, respectively; *d* is the distance between the transmitter and the receiver; λ is the wavelength; and $G_{t}$ and $G_{r}$ are the transmitter and receiver antenna gains, respectively.

The free-space model is a theoretical model, where the path loss ${P\;L}_{F}$ is only determined by the distance and frequency. The fundamental assumption behind the free-space model is that there are no obstacles between or beside the transmitter and receiver. Therefore, the free-space model is considered an extremely optimistic model.

The two-ray (plane earth) model considers both the direct path and the ground reflected waves [22]. Assuming the antenna heights are small compared with the total path length, the plane earth loss can be expressed in decibels as

(2) $${P\;L}_{PE}\;=\;40\log_{10}\left(d\right)-20\log_{10}\left(H_{r}\right)-20\log_{10}\left(H_{t}\right)$$

where $H_{t}$ and $H_{r}$ are defined as the height of the transmitting and receiving antenna, respectively.

In comparison to the free-space model, the two-ray model is more practical because it takes the reflection path and the ground characteristics into account. However, it still makes a few assumptions and simplifications that are somewhat unrealistic. For example, the model only holds for long distances and for cases where the amplitude and phase of the reflected wave is very close to −1.

Factors that affect the radio propagation are very complex. Environmental clutter, obstacles on the propagation path, and all reflection and scattering from other objects can lead to random variations in path loss. As a result, path loss at different locations with the same distance to the transmitter can differ considerably. It is difficult to use a generalized formula or a single model to accurately describe the path loss in various environments. Accordingly, using statistical models becomes an appropriate alternative to express these non-deterministic characteristics of path loss. The log-normal shadowing model is the most commonly used model to characterize these non-deterministic effects. The one-slope log-normal model can be expressed as

(3) $$P\;L\left(d\right)\;=\;P\;L\left(d_{0}\right)+10n\log_{10}\left(\frac{d}{d_{0}}\right)+X_{\sigma }$$

where PL(d) is the average path loss at the distance *d* (expressed in dB) and $P\;L(d_{0})$ is the path loss at a reference distance $d_{0}$ in the vicinity of the transmitter, which can be determined by measurements at $d_{0}$ or the free-space path loss at $d_{0}$. In this study, $d_{0}$ was set to 1 m in consideration of the near-ground WSN scenario. *n* denotes the path loss exponent. $X_{\sigma }$ is a zero mean log-normally distributed random variable with σ standard deviation in dB, expressing the uncertainty caused by shadow fading.

In contrast to the one-slope model, the two-slope log-normal model divides the distance into near field and far field by a breakpoint. The two-slope model can be expressed as

(4) $$P\;L\left(d\right)\;\;=\;\;\left\{\begin{array}{cc} P\;L\left(d_{0}\right)\;+\;10n_{1}\log_{10}\left(\frac{d}{d_{0}}\right)\;+\;X_{\sigma 1}\; & \;(d\leq d_{b})\\ P\;L\left(d_{b}\right)\;+\;10n_{2}\log_{10}\left(\frac{d}{d_{b}}\right)\;+\;X_{\sigma 2}\; & \;(d>d_{b}) \end{array}\right.$$

where $d_{b}$ is the distance between the transmitter and the breakpoint. The breakpoint is the location where the obstruction pierces through the first Fresnel zone. The horizontal separation $d_{b}$ is given by

(5) $$d_{b}\;=\;\frac{1}{\lambda }\sqrt{{\left(\sum^{2}\;-\;\Delta^{2}\right)}^{2}\;-\;2\left(\sum^{2}\;+\;\Delta^{2}\right)\;{\left(\frac{\lambda }{2}\right)}^{2}\;+\;{\left(\frac{\lambda }{2}\right)}^{4}}$$

where λ is the wavelength, $\sum \;=\;H_{t}+H_{r}$ and $\Delta \;=\;H_{t}-H_{r}$.

In addition to the previously mentioned models, there are many other empirical or semi-empirical path loss models, such as the COST231-Hata model and the COST231–Walfish–Ikegami model [23]. However, these models require the antenna to be placed higher than 1 m or even 10 m above the ground. Although these models are widely used in cellular networks, they are not suitable for WSNs in most cases.

## 3. Measurement Setup

### 3.1. Hardware Setup

The designed measurement system mainly consists of two wireless sensor nodes, one as the transmitter and the other as the receiver. A laptop, connected to the receiving node, calculates the path loss by extracting the RSSI from received data packets. We adopted Silicon Labs Si4432 as the radio frequency (RF) chip, and MSP430F5438 as the Microprogramed Control Unit (MCU) chip (Texas Instruments, Dallas, TX, USA). The sensor nodes were custom-built in-house, with the transmitter and receiver nodes working at the 470 MHz band, the data transfer rate set at 9.6 kbps, the transmitting power of +20 dBm, and the antenna gain of 0 dBi.

### 3.2. Measurement Scenarios

**Figure 1:**
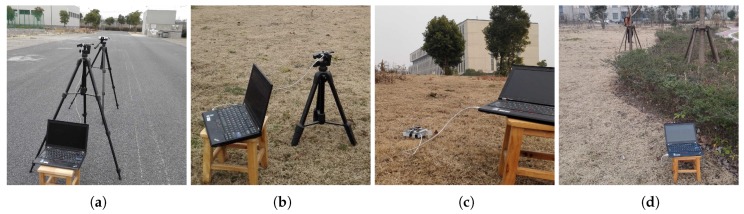
Measurement scenarios: (**a**) flat concrete road; (**b**) flat grass; (**c**) undulating grass; and (**d**) flat grass with obstacles.

Two primary measurement scenarios were selected, which can be described as a flat concrete road and flat grass. The flat concrete road scenario was a straight cement concrete road, which was fairly flat and wide. There were several trees and buildings at the end of the road, but they were rather far away from the measurement field. The flat grass scenario was a nearly flat large lawn, with sparsely scattered bushes and trees nearby. Moreover, two derived scenarios were measured to further investigate the effect of other terrain factors. In the “undulating grass” scenario, the surrounding environment was similar to that of “flat grass”, except that the grass was on wavy terrain. Under this scenario, the transmitter and receiver could only have non-line-of-sight (NLOS) communication unless they were adjacent to each other. The “flat grass with obstacles” scenario was flat grass with bushes between two nodes. Figure 1 shows the four scenarios.

### 3.3. Measurement Methods

The signal strength information can be captured by extracting RSSI from received packets. Based on the conversion relation, the received power can be estimated. According to the typical WSN applications, three different antenna heights were selected (5 cm, 50 cm and 1 m, respectively), among which the 5 cm antenna height represented the situation that sensor nodes were directly thrown to the ground. The transmitter was fixed at one position and the receiver was initially placed at 1 m away from the transmitter. The RSSI was first collected every meter at a distance of up to 10 m from the transmitter, then every 2 m at a distance of up to 20 m, and finally every 5 m until a distance up to 50 m. Hence, there were 21 test points in each measurement. At each test point, 200 packets were collected, providing an adequately large dataset for estimating the statistical properties of path loss. The eventual RSSI value at each test point was an average of the 200 RSSI samples. The same methodology was adopted for each of the above-mentioned scenarios.

We assumed that, in these scenarios, the radio channel was quasi-static. Furthermore, to ensure a fair comparison, the measurement devices were kept stationary by not allowing any moving objects surrounding the test site. In addition, we took the measurement under the same weather condition.

## 4. Measurement Results and Analysis

### 4.1. Measurement Results and Modeling

The relation between transmitted power and received power can be expressed as

(6) $$P_{r}\;=\;P_{t}-P\;L\left(d\right)$$

In the actual experiment, the transmitted power $P_{t}$ was fixed at +20 dBm. Since the received power $P_{r}$ can be derived by extracting RSSI values from the received packets, the path loss PL(d) at distance *d* can be calculated by Equation (6). After completing the data collection, the least square fitting was used to estimate the path loss exponent *n* and correction parameters $X_{\sigma }$, thus the path loss model could be obtained from Equation (3) or Equation (4).

**Figure 2:**
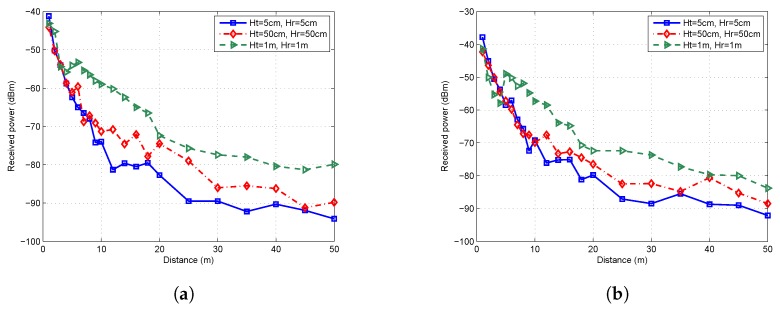
Received power with different antenna heights: (**a**) flat concrete road; and (**b**) flat grass.

The received power in flat concrete road scenario and flat grass scenario are shown in Figure 2a,b, respectively. In general, the received power decayed with the increase of distance. The signal strength decreased more quickly closer to the transmitter, and much more slowly when far away from the transmitter. We also observed that, when the height of the transmitting antenna $H_{t}$ = 5 cm and the height of the receiving antenna $H_{r}$ = 5 cm, the received power was significantly less than that when $H_{t}$ = 1 m and $H_{r}$ = 1 m. In flat concrete road scenario, the received power decayed fairly quickly when $H_{t}$ = $H_{r}$ = 5 cm, with the received power dropping to under −80 dBm at a distance of 12 m from the TX node. However, when $H_{t}$ = $H_{r}$ = 1 m, the received power still reaches −80 dBm at a distance 50 m away from the TX node. A similar trend was observed in the flat grass scenario. Although the values of received power may fluctuate within a certain distance, the overall trend was rather obvious.

**Figure 3:**
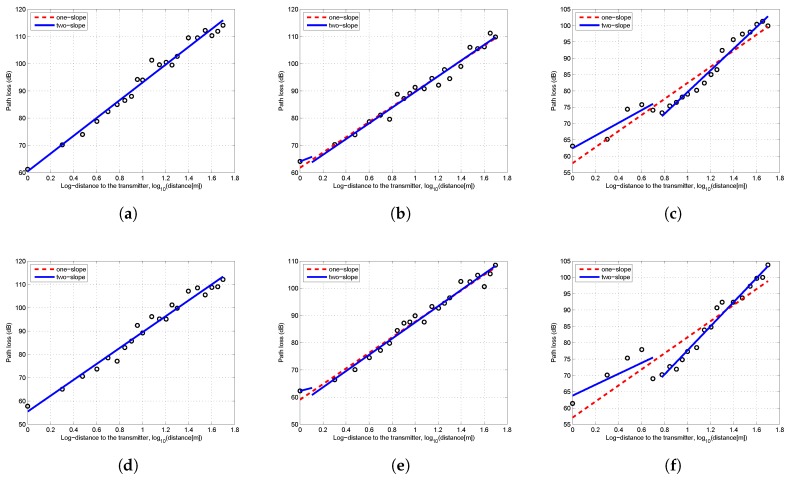
Least squares fitting of measurement results: (**a**) flat concrete road, $H_{t}$ = $H_{r}$ = 5 cm; (**b**) flat concrete road, $H_{t}$ = $H_{r}$ = 50 cm; (**c**) flat concrete road, $H_{t}$ = $H_{r}$ = 1 m; (**d**) flat grass, $H_{t}$ = $H_{r}$ = 5 cm; (**e**) flat grass, $H_{t}$ = $H_{r}$ = 50 cm; and (**f**) flat grass, $H_{t}$ = $H_{r}$ = 1 m.

With the received power data, the path loss can be calculated by Equation (6). Since a log-normal attenuation pattern of the received power can be observed in Figure 2, both the one-slope and two-slope log-normal models were adopted to fit the path loss data. The breakpoint was calculated by Equation (5). The results of least square fitting are shown in Figure 3. When $H_{t}$ = $H_{r}$ = 5 cm, the position of breakpoint was less than 1 m from the transmitter. In that case, the least squares fitting results of one-slope and two-slope models overlap, hence only one line can be seen in Figure 3a,b.

**Figure 4:**
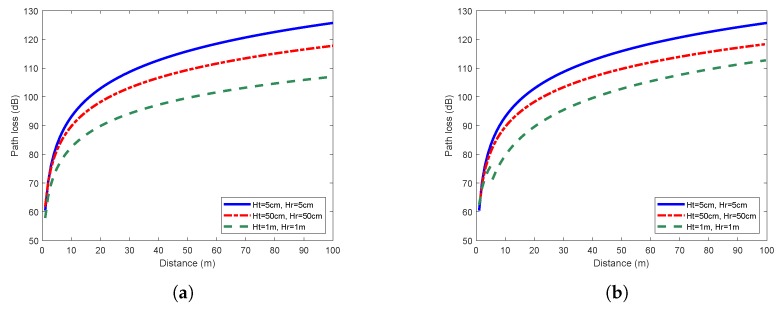
Path loss models under the flat concrete road scenario: (**a**) one-slope model; and (**b**) two-slope model.

**Figure 5:**
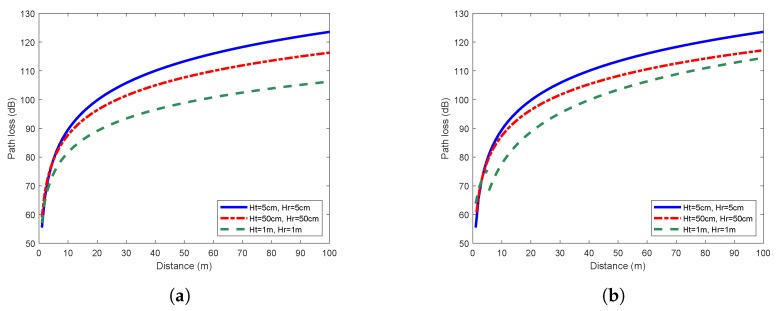
Path loss models under the flat grass scenario: (**a**) one-slope model; and (**b**) two-slope model.

According to the fitting results, the path loss exponent *n* and the random variable $X_{\sigma }$ can be determined, thus we obtained the log-normal shadowing path loss models. Figure 4 shows the path loss models under the flat concrete road scenario. The received power decayed rapidly in the first 20 m, and then declined gradually. In general, the higher was the antenna height, the lower was the propagation loss of the link. This feature was especially noticeable when we compared the path losses with 5 cm and 1 m antenna heights. Figure 5 shows the similar characteristics in the flat grass scenario.

We also tried to fix $H_{t}$ to 5 cm, and vary $H_{r}$ from 5 cm to 1 m. The pattern described above could still be clearly observed, i.e., higher receiving antenna led to higher received power. The antenna height is a key factor affecting the wireless link quality and the signal transmission distance. Therefore, when a WSN is deployed, increasing the antenna height is an effective method to obtain higher received power and gain better coverage. Even if the sensor nodes have to be randomly thrown to the ground in some specific applications, it is still beneficial to increase the antenna height of the sink node.

As shown in Figure 3, as the antenna height of transceivers increased, the difference between the one-slope and two-slope model became more and more obvious. The detailed fitting results are shown in Table 1. When $H_{t}$ = $H_{r}$ = 5 cm, the one-slope model was identical to the two-slope model. Since the one-slope model is simpler and easier to implement, it is more practical to be adopted for scenarios with extremely low antenna height. However, when the antenna height reached 50 cm or higher, there were evident differences between these two models. In the two-slope model, the path loss exponent of the second slope $n_{2}$ was significantly larger than $n_{1}$. The higher was the antenna height, the more obvious was this trend. Furthermore, the precision of both models were obtained by the comparison of values predicted by models and the measured values. The mean error, root mean squared error (RMSE) and mean absolute percentage error (MAPE) of two-slope model were all smaller than those of the one-slope model. This suggests that it is better to adopt the two-slope model to describe the characteristics of near-ground propagation except for the scenario of extremely low antenna height. The low fitting errors shown in Table 1 also validated the effectiveness of using the log-normal shadowing model, which better reflected the real near-ground propagation characteristics.

### 4.2. Comparisons of Propagation Models

**Figure 6:**
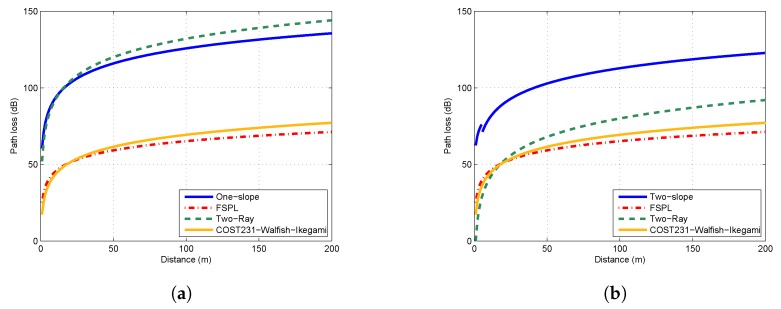
Comparison of path loss models in the flat concrete road scenario: (**a**) $H_{t}$ = $H_{r}$ = 5 cm; and (**b**) $H_{t}$ = $H_{r}$ = 1 m.

Figure 6a,b show the comparison of measured model, free-space path loss model, two-ray model and COST231–Walfish–Ikegami model in the flat concrete road scenario with 5 cm and 1 m antenna heights, respectively. The one-slope model was applied when the antenna height was extremely low (5 cm), while the two-slope model was adopted otherwise. As shown in Figure 6a, the path loss values predicted by FSPL model and COST231–Walfish–Ikegami model were much lower than the values predicted by the one-slope model. This indicated that the wireless channel in near ground was far from a free space. In contrast, the two-ray model was close to the one-slope model in this scenario. However, when antenna heights varied to 1 m, the two-ray model became fairly inaccurate. The path loss predicted by two-ray model varied drastically with the change of antenna height. It can be concluded that these three models are not suitable to represent the characteristics of near-ground channels, due to their unrealistic assumptions and simplifications. The adoption of these models may lead to overestimation or underestimation of path loss.

### 4.3. The Impact of Different Terrains

The measured received power values in Figure 2 indicated that WSNs deployed in concrete road scenario and grass scenario had different transmission distances. Furthermore, other terrain factors may also have an impact on propagation characteristics of wireless channel, resulting in an influenced path loss. To investigate the effects of other terrain factors, two more scenarios were measured, which can be described as “undulating grass” and “flat grass with obstacles”, as shown in Figure 1c,d. In this comparison test, the antenna height of transceivers was fixed to 5 cm.

**Figure 7:**
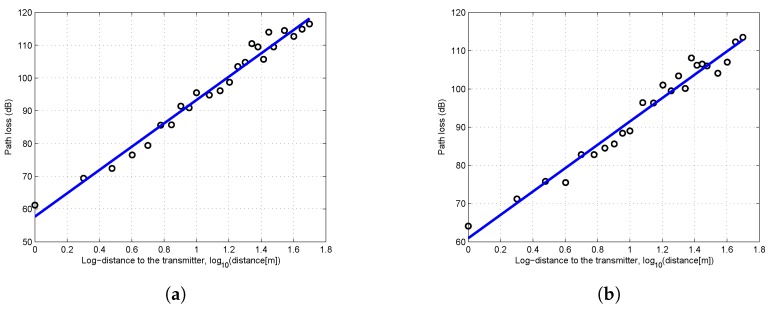
Least squares fitting of measurement results in two derived scenarios: (**a**) undulating grass, $H_{t}$ = $H_{r}$ = 5 cm; and (**b**) flat grass with obstacles, $H_{t}$ = $H_{r}$ = 5 cm.

**Figure 8:**
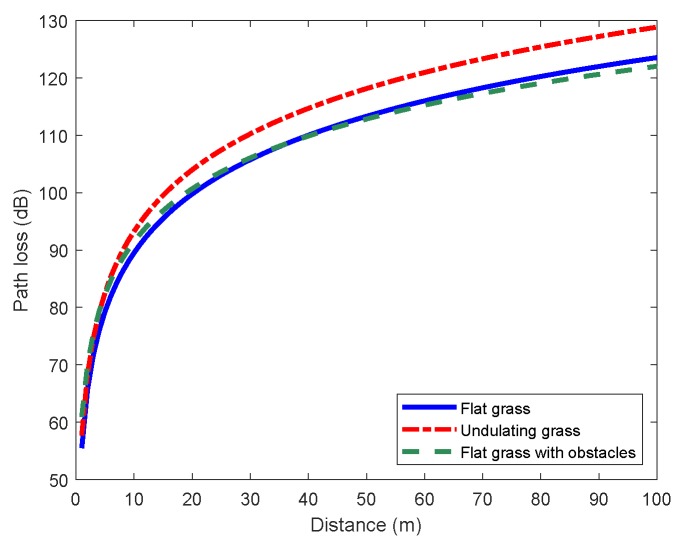
Path loss models with different terrain factors ($H_{t}$ = $H_{r}$ = 5 cm).

Path loss models of these two derived grass scenarios were obtained from the fitting results in Figure 7. Figure 8 shows path loss models of the three grass scenarios. The path loss of undulating grass scenario was visibly greater than other two scenarios. The received power was about 6 dB lower than RX power of flat grass scenario when the distance reached 50 m. This illustrates that the NLOS propagation significantly decayed the signal strength and the received power. On the other hand, there was no obvious difference on path loss between flat grass scenario and flat grass with obstacles scenario, suggesting that signals could transmit through low shrubs without too much attenuation. These results can guide the deployment of the sensor network. When the terrain is undulating, or there are some huge obstacles, it is necessary to add more relay nodes to ensure the reliability of transmission.

**Figure 9:**
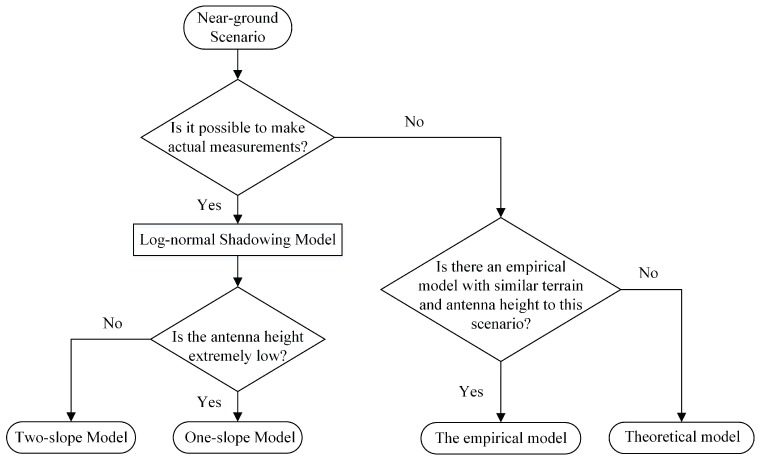
Schematic diagram of the propagation model selection strategy.

Based on the above measurements and analysis, we summarize the propagation model selection strategy in different scenarios in Figure 9. When we need to build a propagation model in a new scenario, the first question is whether we can actually measure the propagation characteristics of this scenario. Terrain and environmental factors can have a large impact on the propagation model, thus only the measured model is the most accurate. Through the actual measurement, we can derive the log-normal shadowing model. It is better to adopt the two-slope model than the one-slope model unless the antenna height is extremely low (e.g., lower than 50 cm). In some cases, it is dangerous or difficult to make actual measurements. At this time, one should first look for an empirical model with similar terrain and antenna height to the scenario, which can obtain predictions that are closer to the actual propagation characteristics. If such a model is not found in previous work, then using a theoretical model such as the two-ray model is also an alternative.

## 5. Simulation and Performance Analysis

Network simulation results are heavily influenced by the selected propagation model, however, most of existing simulators adopt simplistic radio channel models. To further investigate the impact of near-ground propagation on network performance, the implementation of propagation models on a simulator is required. We adopted Cooja [19] as the simulation platform, which is a flexible Java-based simulator designed for WSNs running Contiki OS [24]. Cooja simulates sensor networks where each node can be of different type in terms of software and hardware. The simulated node platform was Tmote Sky (also known as TelosB). We used the Cooja simulator along with the MSPsim emulator [25], which provided accurate emulation in both cycle-level of the MSP430 micro-controller and bit-level of the CC2420 radio transceiver.

We edited the simulation script and added the previously measured path loss models to Cooja. This enabled us to observe the network performance under actual scenarios.

Differences in connectivity have a significant influence on the network communication. Many routing protocols are designed based on the assumption that a node has an exact knowledge of its neighbors. We tested the influence of different propagation models in network connectivity. The simulation scenario was the flat concrete road, with 5 cm height of transmitting and receiving antennas. The deployment area was 200 m × 200 m, the transmitting power was +20 dBm, and the receive sensitivity was −100 dBm. We placed 50, 100, 150, and 200 nodes randomly over the deployment area, respectively, and calculated the average number of connectable neighbors in the transmission range of each node.

**Figure 10:**
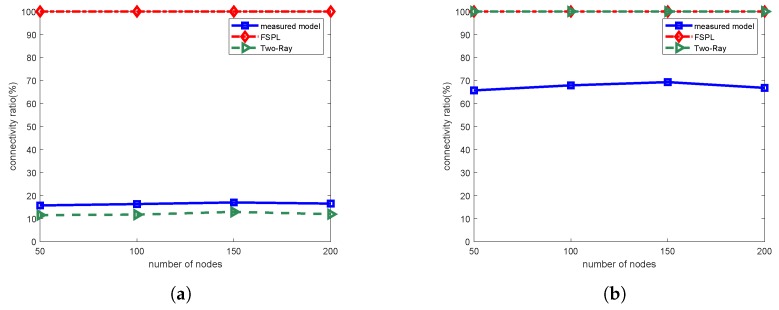
Average percentage of connectivity: (**a**) $H_{t}$ = $H_{r}$ = 5 cm; and (**b**) $H_{t}$ = $H_{r}$ = 1 m.

The average percentage of connectivity is shown in Figure 10, expressed as the average ratio of the number of connectable neighbors to the total number of nodes. When the antenna height was 1 m, the connectivity ratio was obviously higher than the situation that when the antenna height was 5 cm. It is worth noting that, as the antenna height increased from 5 cm to 1 m, the path loss of two-ray model decreased significantly, and the connectivity ratio increased from about 15% to 100%. This indicates that, in near-ground scenarios, the two-ray model is extremely sensitive to antenna height, thus the path loss varies drastically with the change of antenna height. Therefore, the two-ray model has no universal applicability in this scenario. On the other hand, the free-space path loss model produced unrealistic value of path loss: the connectivity ratio remained 100% during the whole simulation, and, as a result, the transmission range was maintained at a level of over 300 m.

From another perspective, the results of simulation using simplistic theoretical models are far from the actual situation and are basically unusable in practical applications. The network performance and the designed protocol achieved in the simulation cannot be realized in practical applications because the connectivity of the network is significantly degraded in the actual scenario. Therefore, when designing and deploying a sensor network, it is important to use an accurate model based on real propagation characteristics in the simulation.

## 6. Conclusions

To accurately reflect the propagation characteristics of near-ground WSNs, we measured the received RSSI values of near-ground deployed sensor nodes under four terrains, including two derived scenarios with different terrain factors. We established the statistical path loss models, and analyzed the influence of different antenna heights and terrain factors. In near-ground scenarios, when the antenna height is extremely low (e.g., lower than 50 cm), the one-slope model is more practical. As the antenna height increases, the precision of the two-slope model becomes higher. Measurement results also show that simplistic theoretical models may lead to overestimation or underestimation of path loss, and thus result in inaccurate estimation of network connectivity. Based on our analyses, we took the first attempt to propose a propagation model selection strategy in near-ground scenarios. In addition, we implemented these propagation models on the Cooja simulator, demonstrating the huge difference in their impact on network connectivity.

## Figures and Tables

**Table 1:** Model parameters and precision.

Model	Item	Flat Concrete Road			Flat Grass		
		5 cm	50 cm	1 m	5 cm	50 cm	1 m
One-slope	*n*	3.264	2.799	2.458	3.403	2.860	2.458
	Mean error	−0.0147	−0.0139	−0.0031	−0.0086	−0.0005	−0.0169
	RMSE	2.0727	2.0355	3.1902	2.4529	2.0175	4.4828
	MAPE	1.75%	1.73%	3.62%	2.25%	2.06%	5.20%
Two-slope	$n_{1}$	–	1.637	1.946	–	1.059	1.666
	$n_{2}$	3.264	2.881	3.315	3.403	2.972	3.685
	Mean error	−0.0147	−0.0104	0.0015	−0.0086	−0.0129	−0.0104
	RMSE	2.0727	1.9448	1.7856	2.4529	1.8368	2.4192
	MAPE	1.75%	1.59%	1.82%	2.25%	1.62%	2.31%

## References

[1] Akyildiz I.F., Su W., Sankarasubramaniam Y., Cayirci E. (2002). Wireless sensor networks: A survey. Comput. Netw..

[2] Younis M., Akkaya K. (2008). Strategies and techniques for node placement in wireless sensor networks: A survey. Ad Hoc Netw..

[3] Karedal J., Wyne S., Almers P., Tufvesson F., Molisch A.F. (2007). A Measurement-Based Statistical Model for Industrial Ultra-Wideband Channels. IEEE Trans. Wirel. Commun..

[4] Ahmed N., Kanhere S.S., Jha S. (2013). Utilizing Link Characterization for Improving the Performance of Aerial Wireless Sensor Networks. IEEE J. Sel. Areas Commun..

[5] Tang W., Ma X., Huang J., Wei J. (2016). Toward improved RPL: A congestion avoidance multipath routing protocol with time factor for wireless sensor networks. J. Sens..

[6] Torabi A., Zekavat S.A. A Rigorous Model for Predicting the Path Loss in Near-Ground Wireless Sensor Networks. Proceedings of the 2015 IEEE 82nd Vehicular Technology Conference (VTC2015-Fall).

[7] Foran R.A., Welch T.B., Walker M.J. Very near ground radio frequency propagation measurements and analysis for military applications. Proceedings of the IEEE Military Communications.

[8] Joshi G.G., Dietrich C.B., Anderson C.R., Newhall W.G., Davis W.A., Isaacs J., Barnett G. (2005). Near-ground channel measurements over line-of-sight and forested paths. IEEE Proc. Microw. Antennas Propag..

[9] Martinez-Sala A., Molina-Garcia-Pardo J.M., Egea-Ldpez E., Vales-Alonso J., Juan-Llacer L., Garcia-Haro J. (2005). An accurate radio channel model for wireless sensor networks simulation. J. Commun. Netw..

[10] Wang D., Song L., Kong X., Zhang Z. (2012). Near-ground path loss measurements and modeling for wireless sensor networks at 2.4 GHz. Int. J. Distrib. Sens. Netw..

[11] Rodriguez M., Feick R., Carrasco H., Valenzuela R., Derpich M., Ahumada L. (2012). Wireless Access Channels with Near-Ground Level Antennas. IEEE Trans. Wirel. Commun..

[12] Kurt S., Tavli B. Propagation model alternatives for outdoor wireless sensor networks. Proceedings of the 2013 IFIP Wireless Days (WD).

[13] Sangodoyin S., Niranjayan S., Molisch A.F. (2016). A Measurement-Based Model for Outdoor Near-Ground Ultrawideband Channels. IEEE Trans. Antennas Propag..

[14] Alsayyari A., Kostanic I., Otero C.E. An empirical path loss model for Wireless Sensor Network deployment in a concrete surface environment. Proceedings of the 2015 IEEE 16th Annual Wireless and Microwave Technology Conference (WAMICON).

[15] Olasupo T.O., Otero C.E., Olasupo K.O., Kostanic I. (2016). Empirical path loss models for wireless sensor network deployments in short and tall natural grass environments. IEEE Trans. Antennas Propag..

[16] Cheffena M., Mohamed M. (2017). Empirical Path Loss Models for Wireless Sensor Network Deployment in Snowy Environments. IEEE Antennas Wirel. Propag. Lett..

[17] Wang J., Dong W., Cao Z., Liu Y. (2015). On the delay performance in a large-scale wireless sensor network: Measurement, analysis, and implications. IEEE/ACM Trans. Netw..

[18] Raheemah A., Sabri N., Salim M.S., Ehkan P., Ahmad R.B. (2016). New empirical path loss model for wireless sensor networks in mango greenhouses. Comput. Electron. Agric..

[19] Osterlind F., Dunkels A., Eriksson J., Finne N., Voigt T. Cross-Level Sensor Network Simulation with COOJA. Proceedings of the 2006 31st IEEE Conference on Local Computer Networks.

[20] Tse D., Viswanath P. (2005). Fundamentals of Wireless Communication.

[21] Saunders S., Aragón-Zavala A. (2007). Antennas and Propagation for Wireless Communication Systems.

[22] Kurt S., Tavli B. (2017). Path-Loss Modeling for Wireless Sensor Networks: A review of models and comparative evaluations. IEEE Antennas Propag. Mag..

[23] Anglès-Vázquez A., Vilajosana-Guillèn X., López-Vicario J., Morell-Pérez A., Tuset-Peiró P., Vilajosana-Guillèn I. (2014). Generic empiric propagation model for low power wireless networks operating at the 868 MHz band in smart cities. Antennas Propag. IET Microw..

[24] Dunkels A., Gronvall B., Voigt T. Contiki—A lightweight and flexible operating system for tiny networked sensors. Proceedings of the 29th Annual IEEE International Conference on Local Computer Networks.

[25] Eriksson J., Dunkels A., Finne N., Osterlind F., Voigt T. Mspsim—An extensible simulator for msp430-equipped sensor boards. Proceedings of the European Conference on Wireless Sensor Networks (EWSN), Poster/Demo Session.

